# A digital diagnostic pathway for heart failure: an economic evaluation

**DOI:** 10.1186/s12962-025-00695-9

**Published:** 2026-01-06

**Authors:** Nicola Mcmeekin, Andrew Davies, Mark C. Petrie, Ross T. Campbell, David J. Lowe, Clare L. Murphy, Leeanne Macklin, Katriona Brooksbank, Olivia Wu

**Affiliations:** 1 Health Economics and Health Technology Assessment (HEHTA), University of Glasgow, Glasgow, UK; 2 School of Cardiovascular and Metabolic Health, University of Glasgow, Glasgow, UK; 3 Queen Elizabeth University Hospital, NHS Greater Glasgow and Clyde, Glasgow, UK; 4 Forth Valley Royal Hospital, NHS Forth Valley, Glasgow, UK; 5 School of Health and Wellbeing, University of Glasgow, Clarice Pears Building, 90 Byres Road, Glasgow, G12 8TB UK

**Keywords:** Economic evaluation, Heart failure, Diagnostic pathways, Heart failure diagnosis, Cost-consequence analysis, Cost-effectiveness

## Abstract

Guidelines recommend prompt diagnosis of suspected heart failure in people with elevated N-terminal pro b-type natriuretic peptide (NT-proBNP). Early diagnosis of heart failure improves patient outcomes, however diagnosis is complex and can often be delayed. Digital platforms can provide clinicians with greater access to information with which to diagnose heart failure, improving on standard diagnostic pathways. We modelled the potential cost-effectiveness of a digital pathway, adapting the UK NICE guideline (NG106) cost-effectiveness model to evaluate the impact of potentially speedier diagnosis and treatment of heart failure. Posited reductions in time to treatment were modelled to reduce short-term mortality and generate approximately 0.056 incremental quality adjusted life-years over a lifetime horizon. Additional costs arose from earlier heart failure treatment, resulting in a cost per quality adjusted life-year of £5,882 (95% confidence interval £4,377 - £11,084).

## Introduction

Early diagnosis and treatment of heart failure (HF) can slow disease progression, avoid hospitalisations, and improve outcomes [1]. European and UK guidelines recommend the use of an N-terminal pro b-type natriuretic peptide (NT-proBNP) blood test for patients with suspected HF in the community, to determine whether and how urgently patients should be referred for standard transthoracic echocardiogram (TTE) for confirmatory diagnosis [2]. The National Institute for Health and Care Excellence (NICE) guideline NG106 for *‘Chronic heart failure in adults: diagnosis and management’* recommends that all patients with NT-propBNP ≥ 400pg/ml should be referred for diagnosis. Patients with NT-propBNP < 2000pg/ml should be treated as routine referrals and seen within six weeks, while those with NT-proBNP > 2000pg/ml are considered urgent and seen within two weeks [3]. The guideline included an economic analysis that found that a NT-proBNP threshold of 400pg/ml is cost-effective. Though the guideline promotes timely intervention, diagnosing HF is complex, there is heterogeneity in HF diagnostic pathways within and between regions, and diagnosis is often delayed; as a result, prognosis for people with HF is poor. A recent meta-analysis reported survival rates at one, five and ten years to be 87%, 57% and 35%, respectively [4]. In practice, patients can endure repeated visits to primary care and hospitals before referral, an often difficult and lengthy process.

Recent advances in digital health technologies include digital platforms and artificial intelligence [5] which have the potential to improve the HF diagnostic pathway. Digital pathways are electronic computerised systems designed for use by clinicians and patients [6]. These systems enhance clinicians’ ability to review newly acquired patient data remotely, improving patient outcomes by reducing time to diagnosis and treatment initiation, promoting speedier treatment of symptoms, and earlier diagnosis of other morbidities. In addition to improving patient outcomes there are benefits in terms of freeing TTE capacity by avoiding unnecessary referral for TTE, improving equity of access to healthcare across diverse geographical areas, and data collection for audit purposes.

The Optimising a Digital Diagnostic Pathway for Heart Failure in the Community study (OPERA) was a prospective observational study that recruited consecutive patients referred onto a digital pathway for confirmation of HF diagnosis following NT-proBNP testing in primary care between 1 December 2020 and 31 August 2021, across five outpatient sites in Glasgow, UK (Clinical trials NCT04724200). The primary objective of the study was to assess the diagnostic accuracy of a handheld transthoracic echocardiogram (TTE) for HF. The study included the use of a digital pathway that has been reported as having shortened times to diagnosis of HF [7].

We sought to estimate the potential cost-effectiveness of introducing a digitised HF diagnostic pathway from the perspectives of the UK NHS and patients.

## Methods

### Overview

We developed a decision analytic model to compare the introduction of a digital pathway for HF diagnosis with current practice in usual care. The model is informed by that developed for NICE clinical guideline NG106, and we adopted that model’s parameter estimates where relevant, such as for risk of hospitalisation and mortality for untreated HF, and effectiveness of HF medications. Patients enter the model upon referral onto the HF diagnostic pathway following a NT-proBNP test. We modelled costs and outcomes using a cost-consequence analysis (CCA) and a cost-utility analysis (CUA), over 12 months and lifetime horizons, respectively. One-year CCA outcomes included the proportion of patients attending each stage in the diagnostic process, including outpatient visits, hospitalisations, time on medication and until diagnosis, and mortality. Both the Medical Research Council (MRC) guidance on developing and evaluating complex interventions [8] and the National Institute for Health and Care Excellence (NICE) Evidence Standards Framework (ESF) for digital health technologies [9] recognise the relevance of CCA when evaluating complex, multi-component interventions and when other outcomes in addition to quality adjusted life-years (QALYs) are of interest to decision-makers.

### Population

Patients referred from primary care to the HF diagnostic pathway, with symptoms suggestive of new HF and with an elevated NT-proBNP (≥ 400pg/ml).

### Comparator and intervention

The comparator is current practice under a usual care pathway, based on the NICE HF diagnosis and management guideline [3] and informed by healthcare professionals working in HF diagnosis, in particular those involved in the OPERA study. Though the NICE guideline recommends referral within two to six weeks, in practice, these referrals may take substantially longer. The usual care HF diagnostic pathway begins with patients undergoing electrocardiogram (ECG) and TTE performed by a cardiac physiologist. The results of these investigations are subsequently reviewed by a cardiologist. Patients diagnosed with HF with reduced ejection fraction (HFrEF) and HF with preserved ejection (HFpEF) are invited back to secondary care for consultation with a HF clinician to discuss their diagnosis and have treatment initiated. Some patients diagnosed with HFpEF may be referred back to their GP with management advice.

The digital pathway is an optimised pathway, which includes a digital platform and ‘one-stop’ service for diagnosis and treatment initiation, potentially bringing forward the appropriate treatment plan. This is based on the digital pathway implemented during the OPERA study. Patients with raised NT-proBNP results (≥ 400pg/ml) are referred onto the digital pathway. Referral details including blood test results, presentation of symptoms, and patient medical history are transferred from general practice and made available on the digital platform. HF clinicians review these details remotely, at a stage termed Active Clinical Referral Triage (ACRT), within days of referral. Where clinicians suspect HF, patients are invited to attend a one-stop diagnostic service. Patients not suspected to have HF exit the diagnostic pathway. Diagnosis for patients with suspected HF based on ACRT is then made at the one-stop service based on ECG performed by a healthcare support worker and TTE performed by a cardiac physiologist, as would eventually be the case under the usual care pathway. A cardiology nurse specialist (CNS) reviews patient information and the TTE results on the digital platform, and logs a preliminary diagnosis of HFrEF, HFpEF or no HF. Patients are informed of their diagnosis by the CNS, who immediately initiates appropriate HF medication. Subsequently, a cardiologist remotely reviews the CNS preliminary diagnosis and submits an electronic comprehensive management plan to the patient’s GP.

### Model

**Fig. 1:**
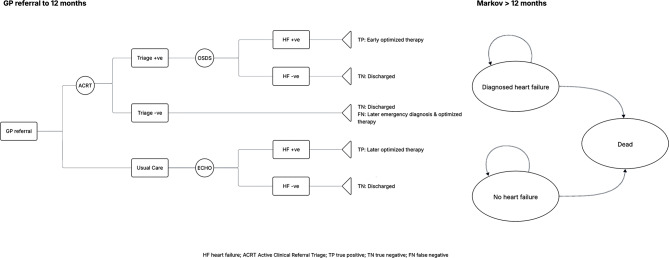
Decision analytic model

A decision analytic model was developed to assess the cost-effectiveness of the digital pathway (Fig. 1). An initial decision tree accounts for outcomes under both pathways over a period of 12 months. Patients then enter a simple long-term Markov process with risks for mortality and heart failure hospitalisation conditional on their 12-month HF status (HFrEF, HFpEF or no HF), over a time horizon of a further 24 annual model cycles. This represents a total time horizon of 25 years, approximating lifetime in this population. The standard UK discount rate of 3.5% is applied to both costs and health outcomes [10]. The model is probabilistic, with relevant parameters entered as appropriate probability distributions and analyses performed over 1,000 iterations. The model was implemented in Microsoft Excel (Microsoft Corporation, 2018. Microsoft Excel, Available at: https://office.microsoft.com/excel).

### Clinical data and assumptions

Patients enter the model with baseline characteristics as observed in OPERA (Table 1), with mean age 75 years (SE 0.359) and 51% female. Most patients had NT-proBNP < 2000pg/ml. The overall prevalence of HF was 81.5%, mainly with HFpEF, and NT-proBNP 400-2000pg/ml.

**Table 1:** Clinical and diagnostic model parameters

Parameter		Value	SD or 95% Confidence Interval	Source
**Patient Characteristics**				
	Age	75	N/A	OPERA
	Female	51% (*n* = 439)	N/A	
NT-proBNP 400–2000 (N, prevalence)	Not HF	80 (18.4%)	N/A	OPERA
	HFrEF	66 (15.2%)	N/A	
	HFpEF	219 (50.3%)	N/A	
NT-proBNP > 2000 (N, prevalence)	Not HF	5 (1.1%)	N/A	OPERA
	HFrEF	24 (5.5%)	N/A	
	HFpEF	41 (9.4%)	N/A	
**Sensitivity | Specificity**	TTE	100% | 100%	N/A	Assumption
	ACRT	100% | 46%	N/A	
**Timing of Pathway Elements**				
Usual care	Referral for TTE	180 days	± 27.0	Assumption
	Cardiologist review	7 days		Assumption
	Cardiologist OPC	49 days		Assumption
Digital pathway	Referral to ACRT	6 days	± 0.9	Assumption
	One-stop service	50 days	± 7.5	OPERA
**Heart Failure Hospitalisations**				
Annual rate of admission	HFrEF	0.219	0.202–0.237	SOLVD
	HFpEF	0.044	0.040–0.049	I_PRESERVE
HR hospitalisation HFrEF *	Standard of Care	0.58	0.475–0.708	EMPHASIS-HF
	SGLT2i	0.70	0.590–0.830	DAPA-HF
HR hospitalisation HFpEF	SGLT2i	0.77	0.670–0.890	DAPA-HF
**Heart Failure Mortality**				
HR Untreated HF vs. population	HFrEF	9.75	6.612–14.369	SOLVD
	HFpEF	2.15	1.461–3.174	I_PRESERVE
Treated HFrEF (versus untreated)*	Standard care	0.44	0.278–0.696	Burnett et al. (2017)
	SGLT2i	0.82	0.690–0.980	DAPA-HF
Treated HFpEF (versus untreated)	SGLT2i	0.91	0.760–1.090	EMPEROR-PRESERVED

Distributions: age/sex: none; NT-proBNP and prevalence: Dirichlet; ACRT specificity: beta; pathway timings: gamma; hazard ratios: lognormal

Clinical inputs for the model are summarised in Table 1. We assumed that none of the patients with HF would be triaged out of the pathway at the ACRT review stage, but allowed for less than 100% sensitivity in scenario analysis. The cost-effectiveness analysis undertaken for the NICE guideline assumed people with HF who went undiagnosed at initial assessment would present as emergencies within six months [3]. We assume, similarly, that such cases will be diagnosed following emergency presentation and standard echocardiographic investigation at 12 months. The total number of patients who entered ACRT was not recorded in OPERA (the study was to assess the diagnostic accuracy of the handheld TTE). 825 patients remained in the diagnostic pathway following ACRT, which we assumed represented 70% of all patients who entered ACRT. Of these, 421 were ultimately confirmed not to have HF. This is equivalent to the assumption that overall, 46% of all patients without HF who entered ACRT would be correctly diagnosed as not having HF.

Empirical data on time between stages in the diagnostic pathways is also not available. The model relies on estimates provided by cardiologists and CNS involved in the OPERA study. In the usual care pathway, the time from referral to TTE is estimated to be 180 days, with a further 56 days for cardiologist review and subsequent cardiologist consultation, meaning treatment plans may not be implemented until almost eight months following initial referral. Under the digital pathway ACRT is estimated to take place within six days. The interval from ACRT to the one-stop diagnostic and treatment initiation service of 50 days that we apply in the model is based on observations in OPERA. Given the uncertainties regarding the accuracy of ACRT in identifying non-HF patients and in usual care timelines, both elements are a key focus in our analyses.

We applied the hospitalisation and heart failure mortality rates based on those used in the economic model reported in NICE guideline. Estimates of untreated HF hospitalisation rates are therefore based on SOLVD for HFrEF [11] and I-PRESERVE for HFpEF [12]. Reductions in these risks were attributed to treatment with standard of care triple therapy for HFrEF based on EMPHASIS-HF [13], with further benefit from treatment with SGLT2i based on DAPA-HF [14], and the EMPEROR preserved trial [15].

Risk of mortality is applied to all patients based on national life tables for Scotland [16], with HF patients modelled to be at elevated risk based on hazard ratios in comparison to the general population depending on ejection fraction, as in SOLVD and I-PRESERVE [11, 12]. Once treatment is initiated, however, risk reductions are applied, again based on EMPHASIS-HF, DAPA-HF, and EMPEROR preserved. Effects in HFrEF are based on DAPA-HF. EMPEROR and DAPA-HF reported similar hazard ratios in HFpEF, and we adopted the more conservative treatment effect for each of hospitalisation and mortality. HF medication confers risk reductions from initiation of treatment. Some patients newly diagnosed with HF may already be treated with HF relevant medication. However, as in the NICE guideline economic model, we assume that this will not be optimised for HF (the diagnosis not having previously been made) and would not impact HF prognosis.

### Health related quality of life

The mean EQ-5D-3L score in OPERA for those ultimately diagnosed with HF was 0.620 (SE 0.014), representing a 17% decrement relative to a UK population norm [17] of 0.755. For patients without HF we applied a UK population norm accounting for reductions with increasing age and maintained the same relative reduction for those with HF throughout the model. Though HF medications have been shown in clinical trials to improve quality of life as measured for example by the Kansas City Cardiomyopathy Questionnaire (KCCQ), we assumed no benefit of HF medication in terms of health-related quality of life. We did, however, apply decrements for HF hospitalisations based on analysis of the SHIFT study [18]. That analysis controlled for SHIFT patients’ NYHA grade and estimated approximately a 0.23 (SE 0.155) reduction in quality of life for HF hospitalisation; whereas the SHIFT analysis applied a decrement over a period 30 days prior to and 30 days after a hospitalisation, we assume a simple 30 days in total for each hospitalisation.

### Resource use

Resource use for each patient referred onto the usual care pathway comprises an ECG and a TTE, an associated cardiologist review (assumed to be ten minutes per review), and a cardiologist out-patient consultation for final diagnosis and treatment initiation. For the digital pathway, we assumed ten minutes cardiologist time per ACRT review, an ECG and a TTE at the one-stop diagnosis service, with 15 min of a healthcare support worker’s time, 45 min of a cardiac physiologist’s time, 45 min of an CNS’s time, and 15 min cardiologist time to remotely review each patient’s results. We also assumed that 25% of patients attending the one-stop service will subsequently require a follow-up consultation with a cardiologist.

Resource use and unit costs are summarised in Table 2. Costs for the digital platform (Lenus Health) include capital costs (integration of IT equipment in pathway), an annual license (which covers 750 patients using the one-stop diagnostic service) and additional user fees (when the number of patients exceeds 750). In the model a unit cost per patient is assigned assuming 1,500 patients are referred to the heart failure diagnosis pathway annually. Other resource use through the pathways were based on standard UK unit cost sources; 2021 costs (£GBP) were applied to resource estimates to calculate the average cost for each pathway. Medication costs are based on British National Formulary [22]. In the case of HFrEF and HFpEF the standard of care four pillar treatment regimen (beta-blockers, aldosterone receptor antagonists valsartan, SGLT2 inhibitors), and SGLT2 inhibitor alone is assumed respectively.

**Table 2:** Unit costs

Resource	Unit cost	Source
Digital platform	£29.05	Lenus Health (digital platform provider)
Active Clinical Referral Triage (ACRT) (10 min cardiologist time)	£20.50	PSSRU 2021 [22]
ECG	£37.00	NHS tariffs 2013/14 [23](uplifted to 2020/21)
Standard transthoracic echocardiogram (TTE)	£145.53	NHS reference costs 2020/21 [21]
One-stop diagnostic service (15 min healthcare support worker, 45 min cardiac physiologist and 45 min cardiology nurse specialist)	£99.80	PSSRU 2021 [22]
Cardiologist review ECG and TTE results (not using digital platform) (10 min cardiologist time)	£20.50	PSSRU 2021 [22]
Cardiologist review of digital platform and complete management plan (15 min cardiologist time)	£30.75	PSSRU 2021 [22]
Clinical consultation with patient for diagnosis and treatment	£257.20	NHS reference costs 2020/21 [24]
Heart failure hospitalisation ⁕	£4,093.01	NHS ref costs 2020/21 [24]
HFrEF treatment⁑ monthly	£96.75	British National Formulary [25]
HFpEF treatment† monthly	£39.75	British National Formulary [25]

⁕ A gamma distribution was assigned for HF hospitalisation (standard error assumed 15% of mean)

⁑HFrEF: Beta blockers − 50% carvedilol 50 mg b.i.d; 50% bisoprolol 10 mg q.d.; aldosterone receptor antagonists (MRA) − 50% spironolactone 50 mg q.d.; 50% eplerenone 50 mg q.d.; sacubitril/valsartan – 97 mg/103 mg q.d.; SGLT2i dapagliflozin or empagliflozin 10 mg q.d

†HFpEF: Sacubitril/valsartan as per HFrEF

### Analysis

We modelled short term (within 12 months) outcomes (as described above), resource use and survival, and lifetime cost per QALY. The main analyses were performed probabilistically. Sensitivity analyses are presented based on the deterministic model.

## Results

Results for short- and long-term analyses are presented in Table 3.

**Table 3:** Outcomes and Cost-effectiveness

	Usual care pathway		Digital pathway	
One-year outcomes				
	Mean Units	£	Mean Units	£
ACRT	-	-	0.998	80
Echo	0.944	192	0.895	163
One-stop service	-	-	0.895	89
Clinic visit (HF clinician)	0.788	202	0.201	52
Sub-total		394		385
Hospitalisation	0.055	225	0.041	171
Time on medication		191		456
Total one-year cost (£)		810 (651–969)		1,011 (920–1,114)
Time to diagnosis (weeks)	32 (26–39)		7 (6–9)	
One-year mortality (%)	0.096 (0.073–0.122)		0.076 (0.056–0.100)	
**Long-term**				
Diagnosis (year 1) (£)		394 (380–406)		385 (377–391)
Hospitalisation (£)		838 (528–1,219)		812 (496–1,211)
Medication (£)		2,063 (1,289–2,892)		2,416 (1,610–3,277)
Total costs (£)		3,295 (2,284–4,420)		3,612 (2,542–4,841)
Life years	6.388 (4.991–7.92)		6.488 (5.00–8.047)	
QALYs	3.577 (2.894–4.344)		3.631 (2.914–4.415)	
Cost-per QALY (£)			5,882 (4,377–11,084)	

### Short term cost-consequence analysis

Over one-year, 89.5% of patients entering the digital pathway were expected to attend the one stop service and undergo echocardiography, compared to an expected 94.4% of patients in the usual care pathway undergoing echocardiography, with 78.8% of patients in the usual care pathway going on to consultation with a HF clinician.

Mean time to diagnosis, under this model, including for those found not to have HR, was 224 days and 51 days for the usual care and digital pathway respectively. For those ultimately diagnosed with HF time to treatment is assumed to be 236 and 56 days respectively. The earlier confirmation of appropriate treatment resulted in higher medication costs for the first year in the digital pathway of £456 compared to £193 for usual care. Earlier treatment resulted in fewer hospitalisations and lower first year mortality of 7.6% for the digital pathway compared to 9.6% for usual care. Total first year costs, including hospitalisations, were £201 higher for the digital pathway.

### Cost-utility analysis

**Fig. 2:**
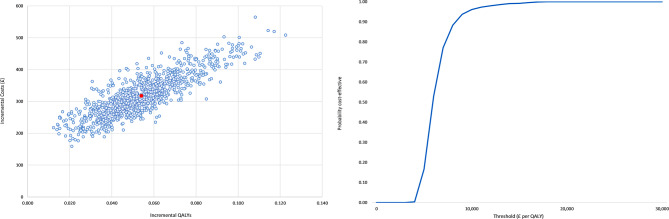
Cost-utility plane and cost-effectiveness acceptability curve

Reduced one year mortality in the digital pathway was extrapolated to lead to a mean increase in (discounted) life expectancy of 0.105 years, or 0.056 QALYs. Greater one year survival also resulted in higher lifetime treatment costs for the digital pathway of approximately £350 per patient. The total incremental lifetime cost was modelled to be £317, with an incremental cost per QALY for the digital pathway of £5,882 (95% confidence interval £4,377-£11,084), with a 100% probability of being cost-effective at a cost-effectiveness threshold of £20,000 per QALY (Fig. 2).

**Fig. 3:**
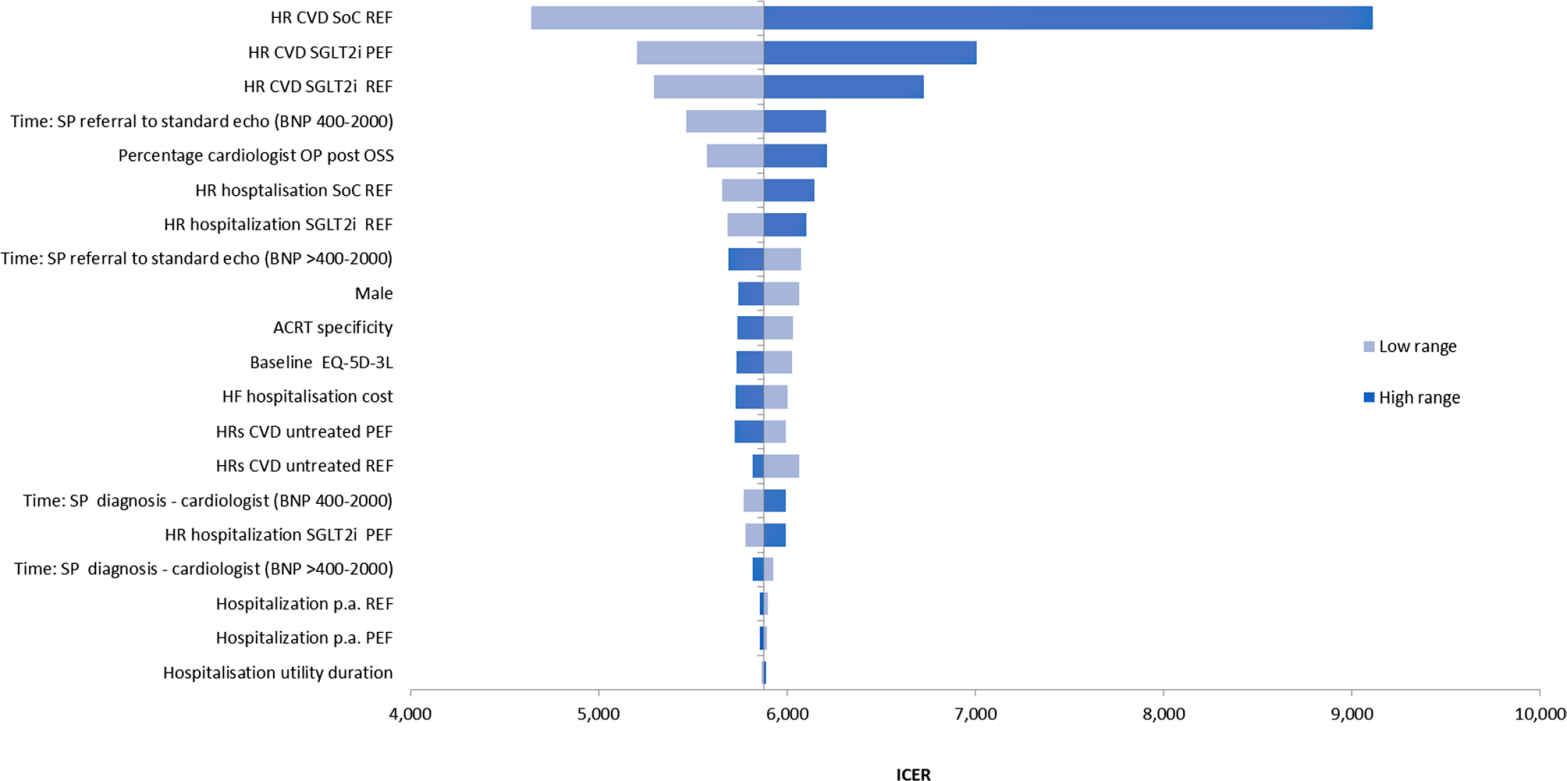
Sensitivity analyses

Sensitivity analysis showed the digital pathway’s cost-effectiveness to be relatively insensitive to replacement of base case parameter values with their 95% confidence ranges (Fig. 3). The digital pathway’s cost-effectiveness was most sensitive to the degree of benefit assigned for HF treatments, with the incremental cost-effectiveness ratio (ICER) reaching £9,110 when the upper 95% confidence interval for standard of care HR for cardiovascular death (CVD) in HFrEF was applied. When the time from referral to standard echo in the usual care pathway was assumed to be 131 rather than 180 days, the digital pathway’s ICER fell marginally to £5,467, as standard care medication costs rose as a result.

In addition to the sensitivity analyses summarised in Fig. 2, we conducted scenario analyses applying alternative sources for medication efficacy, varying the impact on quality of life of HF hospitalisation (± 50%), and varying time from referral to TTE in the standard care arm (-90, + 50 days), and from ACRT to the one stop service (+ 50 days) (Table 4). None of these had any notable impact on the ICER. There is uncertainty relating to the presumed accuracy of the ACRT element within the digital pathway, as discussed above. We applied an estimate for the rate of triage from the digital pathway at ACRT of 30% in the base case analysis. Adopting alternative estimates of 20% and 40% also had negligible impact on the cost per QALY (£5,765 and £6,005 respectively). In the base case we assumed all patients with HF would be retained in the digital diagnostic pathway at the ACRT stage, based on clinician’s understanding that in OPERA there had been no late diagnoses of HF after ACRT triage. When we assumed that 10% and 20% of HF cases might be triaged out of the pathway in error, and be diagnosed later, incremental QALYs were reduced, but cost per QALY rose only slightly (to £5,889 and £5,907 respectively). The greatest impact on cost-effectiveness in scenario analyses was seen when the risk reduction with SGLT2i medication (for both HFrEF and HFpEF), was reduced by 50%, with cost per QALY rising to approximately £7,000.

**Table 4:** Scenario analyses

	Standard			Digital			Incremental			
	Costs (£)	Life years	QALYs	Costs (£)	Life years	QALYs	Costs (£)	Life years	QALYs	CE ratio
ACRT specificity low	3,265	6.348	3.565	3,592	6.449	3.619	327	0.101	0.054	6,005
ACRT specificity high	3,265	6.348	3.565	3,578	6.449	3.619	314	0.101	0.054	5,765
ACRT sensitivity 90%	3,265	6.348	3.565	3,532	6.432	3.610	267	0.084	0.045	5,889
ACRT sensitivity 80%	3,265	6.348	3.565	3,479	6.415	3.601	214	0.067	0.036	5,907
Reduced effectiveness of SGLT2I	3,214	6.104	3.446	3,535	6.188	3.491	321	0.084	0.046	7,045
Hospitalisation utility decrement, -50%	3,265	6.348	3.564	3,585	6.449	3.618	320	0.101	0.054	5,881
Time from referral to standard echo − 90 days	3,434	6.397	3.592	3,585	6.449	3.620	150	0.052	0.028	5,403
Time from referral to standard echo + 50 days	3,172	6.321	3.551	3,585	6.449	3.619	413	0.127	0.069	6,016
Time from ACRT triage to one-stop service − 15 days	3,265	6.348	3.565	3,614	6.457	3.624	349	0.110	0.059	5,895
Time from ACRT triage to one-stop service + 15 days	3,265	6.348	3.565	3,555	6.440	3.615	291	0.092	0.050	5,852

## Discussion

There is growing interest in the adoption of digital health technologies, to tackle increasing pressures on healthcare systems. Healthcare commissioners are faced with early adoption decisions based on limited available evidence. The introduction of digital health technologies to the HF diagnostic pathway is one such example. We sought to align our assumptions closely with those of the NICE economic model for NG106. This allows consistency between NG106 and the current analysis regarding risks for hospitalisation and mortality, both for untreated HF and following implementation of optimised therapy. As in NG106 we found that in both sensitivity and scenario analyses the ICER was insensitive to variations in these elements of the model. Our analysis requires suitable estimates for hospitalisation and mortality, but these apply equally to both the standard diagnostic pathway and the proposed digital alternative. The cost-effectiveness of the latter derives from earlier diagnosis and hence optimised therapy. We assumed effectiveness of medical therapy based on key clinical studies as in NG106 and applied these treatment effects from the moment of first post-diagnosis prescription. In practice gaining the full benefit of these therapies may require follow up appointments to fully optimise therapy, but this would be the case under either pathway, and substantial reductions in the effectiveness of these therapies, while having a greater impact than other more pessimistic scenarios, increased the cost per QALY only marginally.

The OPERA study was a prospective observational cohort study where all patients with suspected HF presenting at primary care were referred to ACRT [7]. The goal of the study was to assess the handheld device in this context. The COVID-19 pandemic severely disrupted the study, and we rely in our model on estimates for likely time to each point in both pathways on the basis of recent clinical experience in Glasgow. The study has been reported to have reduced referrals for heart failure diagnostic tests, including echocardiography, allowed consultants to deliver ten clinical management plans in the time required for four face to face consultations, and reduced heart failure diagnosis waiting times from 12 months to six weeks [7]. We applied a more optimistic assumption of 180 days for diagnosis waiting time under usual care.

This analysis illustrates the potential benefits both for healthcare systems and patients that may be achieved with adoption of a digital pathway. We presented the short-term costs and consequences under the alternative pathways in the manner of CCA, as advocated in complex interventions and digital intervention evaluation guidelines. Quantifying specific short-term outcomes such as consultant time savings and diagnosis waiting times may assist health services commissioners considering the implementation of innovations such as the digital pathway. Both over the short-term, and lifetime analyses, the digital pathway is modelled to be associated with additional total health care costs, however, this is due to the earlier prescription of appropriate medication, and the greater short-term survival as a consequence. Short-term survival is higher in the digital pathway due to earlier diagnosis, however, over a life-time this incremental benefit attenuates, because after one year we assume equal treatment benefits in both pathways, based on the assumption that while the digital pathway accelerates diagnosis, HF is diagnosed with treatment optimized for all patients who survive 12 months, irrespective of the diagnostic pathway. This conforms with the model for NG106, and indeed, under existing referral standards, people previously referred for HF diagnosis who re-present are not eligible for referred to the pathway a second time and must be referred directly to a cardiology clinic.

There are limitations to our study in addition to the current paucity of data regarding time intervals between stages of the diagnostic pathways.

We treated TTE as the gold standard for diagnosis. This is broadly in line with previous published studies [19, 20]. We also assumed perfect sensitivity of TTE plus expert diagnosis (either cardiologist or cardiology nurse specialist) at the ACRT triage stage within the digital pathway. We did so on the basis of firm clinical opinion, but allowed for some false negatives at this stage in scenario analyses. As one would expect, lower sensitivity diminishes the digital pathway’s cost-effectiveness. However, though delays would be incurred relative to a perfect ACRT triage, the relevant comparison remains with delayed diagnosis under the current standard pathway, and we found the pathway’s cost-effectiveness to be robust to reasonable reductions in sensitivity at ACRT.

The value of earlier diagnosis lies in bringing forward treatment plans that include optimised heart failure medication. We based both underlying clinical event rates, and the protective effects of medication on the same evidence adopted in the NICE heart failure management clinical guideline’s economic model [19] assigning benefits from optimised therapy in line with current guidelines. We did not seek more recent evidence. As with reduced sensitivity at ACRT, sensitivity analysis suggested that cost per QALY may be higher than modelled in our base case if the overall effectiveness of treatment in reducing CVD deaths was less than we applied based on SOLVD and I-PRESERVE, but that the digital pathway would remain cost-effective. The NG106 guideline model report acknowledged that some patients were taking HF related medication at baseline in these trials, but that this would have had minimal impact. Fewer than 10% of the participants in the SOLVD trial were taking beta blockers at baseline, and whilst 85% were taking diuretics, the effect of these on reducing mortality were expected to have been relatively little compared to HF standard of care. Moreover, diuretics are not one of the current usual medications for heart failure. The baseline medications the participants in the I-PRESERVE trial were receiving would be for co-morbidities and would not impact heart failure prognosis.

We used general population health utilities in our model to characterise quality of life for people without HF. This could result in some overestimation of quality of life for patients presenting with HF like symptoms whose quality of life may not be as good as the general population. We conducted a range of sensitivity and scenario analyses to test our assumptions and found the digital pathway remained cost-effective across these analyses.

There may be additional benefits associated with the digital platform that our analysis does not consider. In our analysis we assign equivalent risks for hospitalisation and mortality to all treated HF patients. However, the digital pathway extends to virtual clinical management which can involve ongoing collection of data and optimisation of medication based on changing clinical characteristics. This may help avoid hospital admissions through earlier detection of worsening heart failure, preserve health related quality of life, and potentially reduce mortality.

Future involvement of artificial intelligence (AI) in the digital pathway might further improve efficiency of diagnosis [21]. Introducing AI could reduce the number of people without HF referred, reducing the burden on either usual care or diagnostic pathways. Whilst the possible service efficiencies are clear, patient outcomes would depend on the sensitivity and specificity of the AI component used, and the present analysis does not involve any consideration of AI.

## Conclusions

This economic evaluation has estimated the value of a digital pathway, indicating adoption of an OPERA-style digital pathway could be a cost-effective strategy for the NHS and the patients in Scotland. However, there are uncertainties associated with limitations of available data. In order to mitigate risk of investment, decision makers might consider a coverage with evidence approach, where the health technology is adopted for a fixed time period with the requirement for additional data collection and future evaluation.

## Data Availability

Study data and model will be shared on a reasonable request to the corresponding author.
